# Supplementary material for: The indirect health effects of malaria estimated from health advantages of the sickle cell trait
Source: Nat Commun. 2019 Feb 20;10:856. doi: 10.1038/s41467-019-08775-0 (PMC6382840; doi:10.1038/s41467-019-08775-0)
Supplement: Supplementary file 1 — Supplementary Information [file 41467_2019_8775_MOESM1_ESM.pdf]

Supplementary Table 1. The clinical phenotypes of hospital-admitted patients, defined hierarchically.

Syndrome	HbAA N=16,502	HbAS N=1,791
Neonatal conditions	1,615 (9.8)	224 (12.5)
Bacteremia	709 (4.3)	83 (4.6)
Severe malaria	797 (4.8)	17 (1.0)
Malaria	6,223 (37.7)	348 (19.4)
Severe anemia	380 (2.3)	50 (2.8)
Meningitis/encephalitis	973 (5.9)	140 (7.8)
Severe malnutrition	580 (3.5)	82 (4.6)
Very severe pneumonia	3,401 (20.6)	531 (29.7)
Severe pneumonia	133 (0.8)	20 (1.1)
Gastroenteritis	622 (3.8)	92 (5.1)
Other	1,069 (6.5)	204 (11.4)

Supplementary Table 2. Odds ratios for specific hierarchically-defined clinical phenotypes.

Diagnosis	OR (95% CI)*	*P*	aOR (95% CI)*	*P*
Neonatal conditions	0.79 (0.67-0.93)	0.005	0.79 (0.67-0.93)	0.005
Bacteremia	0.67 (0.53-0.85)	0.001	0.70 (0.54-0.91)	0.007
Severe malaria	0.12 (0.07-0.20)	<0.0001	0.12 (0.08-0.20)	<0.0001
Malaria	0.32 (0.28-0.37)	<0.0001	0.31 (0.27-0.37)	<0.0001
Severe anemia	0.75 (0.55-0.98)	0.039	0.75 (0.55-1.04)	0.095
Meningitis/encephalitis	0.82 (0.68-0.99)	0.049	0.83 (0.67-1.04)	0.11
Severe malnutrition	0.81 (0.63-1.03)	0.088	0.81 (0.62-1.06)	0.13
Very severe pneumonia	0.89 (0.79-1.01)	0.066	0.92 (0.79-1.07)	0.27
Severe pneumonia	0.86 (0.53-1.38)	0.53	0.87 (0.53-1.43)	0.59
Gastroenteritis	0.85 (0.67-1.06)	0.16	0.86 (0.66-1.11)	0.24
Other	1.06 (0.91-1.24)	0.46	1.16 (0.99-1.36)	0.072

Crude (OR) and adjusted (aOR) odds ratios derived through comparison of allele frequencies in cases versus community controls by logistic regression, without or with adjustment respectively for gender and division of residence. P-values were estimated using logistic regression.
