# Supplementary material for: The indirect health effects of malaria estimated from health advantages of the sickle cell trait
Source: Nat Commun. 2019 Feb 20;10:856. doi: 10.1038/s41467-019-08775-0 (PMC6382840; doi:10.1038/s41467-019-08775-0)
Supplement: Supplementary file 3 — Reporting Summary [file 41467_2019_8775_MOESM3_ESM.pdf]

nature research

Corresponding author(s): Professor Thomas N Williams

Last updated by author(s): Jan 26, 2019

Reporting Summary

Nature Research wishes to improve the reproducibility of the work that we publish. This form provides structure for consistency and transparency in reporting. For further information on Nature Research policies, see Authors & Referees and the Editorial Policy Checklist.

Statistics

For all statistical analyses, confirm that the following items are present in the figure legend, table legend, main text, or Methods section.

n/a	Confirmed	
☐	☒	The exact sample size (*n*) for each experimental group/condition, given as a discrete number and unit of measurement
☐	☒	A statement on whether measurements were taken from distinct samples or whether the same sample was measured repeatedly
☐	☒	The statistical test(s) used AND whether they are one- or two-sided<br>*Only common tests should be described solely by name; describe more complex techniques in the Methods section.*
☐	☒	A description of all covariates tested
☐	☒	A description of any assumptions or corrections, such as tests of normality and adjustment for multiple comparisons
☐	☒	A full description of the statistical parameters including central tendency (e.g. means) or other basic estimates (e.g. regression coefficient) AND variation (e.g. standard deviation) or associated estimates of uncertainty (e.g. confidence intervals)
☐	☒	For null hypothesis testing, the test statistic (e.g. *F*, *t*, *r*) with confidence intervals, effect sizes, degrees of freedom and *P* value noted<br>*Give P values as exact values whenever suitable.*
☒	☐	For Bayesian analysis, information on the choice of priors and Markov chain Monte Carlo settings
☐	☒	For hierarchical and complex designs, identification of the appropriate level for tests and full reporting of outcomes
☒	☐	Estimates of effect sizes (e.g. Cohen's *d*, Pearson's *r*), indicating how they were calculated

Our web collection on statistics for biologists contains articles on many of the points above.

Software and code

Policy information about availability of computer code

Data collection	The code associated with the statistical analysis of the primary data, written in Stata v11.2, have been deposited on the data repository for the KEMRI/Wellcome Trust Research Programme in Kilifi and are available, along with appropriately anonymized data, by application through <MMunene@kemri-wellcome.org>.
Data analysis	Data analysis was through code written in Stata v11.2

For manuscripts utilizing custom algorithms or software that are central to the research but not yet described in published literature, software must be made available to editors/reviewers. We strongly encourage code deposition in a community repository (e.g. GitHub). See the Nature Research guidelines for submitting code & software for further information.

Data

Policy information about availability of data

All manuscripts must include a data availability statement. This statement should provide the following information, where applicable:

- Accession codes, unique identifiers, or web links for publicly available datasets
- A list of figures that have associated raw data
- A description of any restrictions on data availability

Requests for access to appropriately anonymized data from this study can be made by application to the data access committee at the KEMRI/Wellcome Trust research Progamme through the following e-mail address: <MMunene@kemri-wellcome.org>. The authors declare that all other data supporting the findings of this study are available within the article and its Supplementary Information files, or are available from the authors upon request.

Field-specific reporting

Please select the one below that is the best fit for your research. If you are not sure, read the appropriate sections before making your selection.

☒ Life sciences ☐ Behavioural & social sciences ☐ Ecological, evolutionary & environmental sciences

For a reference copy of the document with all sections, see nature.com/documents/nr-reporting-summary-flat.pdf

Life sciences study design

All studies must disclose on these points even when the disclosure is negative.

Sample size	The sample size for cases was pragmatically determined on the basis of all children presenting to the target hospital over a full five-year period. The sample size for controls was determined pragmatically on the basis of sample availability.
Data exclusions	No data were excluded.
Replication	The sample set is unique and replication is not possible.
Randomization	Participants were consecutive children presenting to the health facility during the period of study.
Blinding	This was a retrospective study. Blinding was not possible or necessary.

Reporting for specific materials, systems and methods

We require information from authors about some types of materials, experimental systems and methods used in many studies. Here, indicate whether each material, system or method listed is relevant to your study. If you are not sure if a list item applies to your research, read the appropriate section before selecting a response.

Materials & experimental systems

n/a	Involved in the study	
☒	☐	Antibodies
☒	☐	Eukaryotic cell lines
☒	☐	Palaeontology
☒	☐	Animals and other organisms
☐	☒	Human research participants
☐	☒	Clinical data

Methods

n/a	Involved in the study	
☒	☐	ChIP-seq
☒	☐	Flow cytometry
☒	☐	MRI-based neuroimaging

Human research participants

Policy information about studies involving human research participants

Population characteristics	Case patients: diagnosis, age, sickle cell genotype, area of residence, date of admission, full blood count, nutrition status.<br>Controls: age, sickle cell genotype, area of residence, date of sampling.
Recruitment	Cases: All children presenting to the central health facility during the period of study. These were self selected on the basis of their clinical status.<br>Controls: Randomly recruited to studies conducted in the same study area and during the same time period as cases. No self selection.
Ethics oversight	Ethical permission for this study was granted by the KEMRI/National Ethics Research Committee in Nairobi, Kenya.

Note that full information on the approval of the study protocol must also be provided in the manuscript.

Clinical data

Policy information about clinical studies

All manuscripts should comply with the ICMJE guidelines for publication of clinical research and a completed CONSORT checklist must be included with all submissions.

Clinical trial registration	This was not a clinical trial.
Study protocol	There is no protocol for this study. This analysis was conducted as a sub-study of two clinical surveillance platforms and was not a stand-alone project.

Data collection	Cases: data collected at the Kilifi County Hospital in Kilifi Kenya between 01 Jan 2000 and 31 Dec 2004.<br>Controls: Data collected from the area served by the Kilifi Health and Demographic Surveillance System surrounding Kilifi District Hospital, Kenya during the same period as cases.
Outcomes	We predefined the primary outcome, death in hospital, and measured it through clinical follow up of all admitted patients.
